# When Chronic Pain Clouds Acute Diagnosis: Femoral Shaft Osteonecrosis With Superimposed *Pseudomonas* Osteomyelitis in a Medically Complex Patient

**DOI:** 10.1002/ccr3.72968

**Published:** 2026-06-17

**Authors:** Ji‐Hoon Lee, Elias Lai, Jerry Markar, Bi Mo

**Affiliations:** ^1^ David Geffen School of Medicine at UCLA Los Angeles California USA; ^2^ Department of Anesthesiology and Perioperative Medicine, Division of Pain Medicine David Geffen School of Medicine at UCLA Los Angeles California USA

**Keywords:** avascular necrosis, chronic systemic steroid, myelodysplastic syndrome, osteonecrosis, *pseudomonas* osteomyelitis

## Abstract

In medically complex patients with chronic pain, new or worsening symptoms should prompt careful reassessment. Avascular necrosis, particularly in the setting of prolonged steroid use, may be overlooked due to diagnostic overshadowing. Timely recognition requires multidisciplinary collaboration and vigilance to distinguish acute pathology from baseline chronic conditions.

## 1 Introduction

Avascular necrosis (AVN), also referred to as osteonecrosis, is a progressive disorder in which disruption of the bone's micro‐circulation precipitates ischemic death of osteocytes, loss of structural integrity, and eventual joint collapse if left untreated [1]. Hip involvement is most frequent—accounting for roughly 40% of the national osteonecrosis burden in the United States [2, 3].

The disease represents a final common pathway for heterogeneous insults. Direct vascular injury (e.g., displaced fracture or dislocation), intravascular obstruction by thrombi or fat emboli, and extravascular compression from marrow‐fat hypertrophy or Gaucher cells all diminish perfusion to the subchondral plate [4]. Glucocorticoid therapy is the single most important non‐traumatic trigger: lipid deposition and micro‐embolization raise intra‐osseous pressure, compromising venous outflow and arterial inflow [5]. Alcohol misuse has a similar lipogenic effect, and together alcohol and corticosteroids are implicated in up to 80% of non‐traumatic cases [6]. Additional predisposing factors include sickle‐cell disease, systemic lupus erythematosus, coagulopathies, heavy tobacco use, HIV infection, prior radiation, and certain myeloproliferative disorders [7].

Osteomyelitis is most commonly caused by *Staphylococcus aureus* ; however, *Pseudomonas aeruginosa* accounts for a minority of cases overall, estimated at approximately 3%–10% depending on the population studied [8]. *Pseudomonas aeruginosa* is classically associated with puncture wounds through footwear [9], intravenous (IV) drug use [10], diabetic foot ulcers [11], immunocompromised hosts [12], postoperative or implant‐associated infections [13], and post‐traumatic osteomyelitis [14]. In contrast, superimposed infection in the setting of osteonecrosis is rarely reported and may be diagnostically challenging because osteonecrosis and infection share overlapping clinical symptoms and radiographic findings, including progressive pain, marrow edema, joint effusion, and structural collapse [15].

Here, we present a case of a 78‐year‐old man with a complicated medical history who developed acute‐on‐chronic left hip pain. He had been treated with high‐dose systemic corticosteroids for presumed autoimmune cytopenia and was later hospitalized multiple times with concern for evolving myelodysplastic syndrome (MDS). His hip pain, initially attributed to chronic baseline arthralgia, persisted and acutely and severely worsened. Advanced imaging and biopsy ultimately confirmed femoral shaft osteonecrosis complicated by *Pseudomonas aeruginosa* osteomyelitis. He was treated with IV antibiotics and remains on chronic oral antibiotics due to ongoing neutropenia.

## 2 Case Presentation

### 2.1 Clinical Course

The patient was a 78‐year‐old male with chronic multifactorial pain, prostate adenocarcinoma in remission following treatment with bicalutamide and leuprolide, atrial fibrillation, hypertension, diabetes mellitus, hyperlipidemia, degenerative disc disease, and progressive pancytopenia concerning for myelodysplastic syndrome (MDS). Surgical history included gynecomastia surgery and cardiac ablation for atrial fibrillation.

Given the complexity of the patient's longitudinal course, key clinical events, diagnostic evaluations, and therapeutic interventions are summarized in Table 1.

**TABLE 1:** Summary of the patient's longitudinal clinical course, including symptom progression, diagnostic evaluations, treatments, and major clinical events leading to the diagnosis of steroid‐associated femoral shaft osteonecrosis complicated by *Pseudomonas aeruginosa* osteomyelitis.

Relative time course	Clinical events	Management
4 years prior to osteomyelitis diagnosis	Developed bilateral lower‐extremity numbness, paresthesias, buttock/groin pain, and multifactorial chronic pain syndrome. Serial lumbar MRIs demonstrated only mild degenerative changes	Physical therapy and conservative pain management
Following years	Persistent multifactorial pain attributed to radiculopathy, sacroiliac dysfunction, hip pathology, and piriformis syndrome	Managed with opioid analgesics, including hydrocodone‐acetaminophen 10/325 mg BID PRN and transdermal buprenorphine 15 mcg/h patch, as well as multiple image‐guided steroid injections
8 months prior to presenting acute hip pain	Initial ED presentation with fatigue and body aches; laboratory evaluation revealed pancytopenia. Initial bone marrow biopsy showed no clonal or infiltrative process	Prednisone 30 mg QAM initiated for presumed autoimmune etiology
Following 2 months	Prednisone adherence was inconsistent for approximately 2 months before resumption; CBC improved after restarting therapy	Prednisone tapered to 20 mg, then 10 mg QAM; later increased to 15 mg QAM for persistent fatigue
~3 months after initial ED visit	Concern for evolving MDS	Rituximab initiated with prednisone 20 mg QAM; total of four rituximab infusions administered
Subsequent months	Returned to ED with palpitations; inpatient workup was non‐revealing. Two months later, returned with anemic symptoms; repeat bone marrow biopsy showed 5%–10% myeloblasts. He was later hospitalized for gastrointestinal bleeding and then DVT requiring IVC filter placement	Prednisone had been tapered to 4 mg QAM by the anemia presentation; received 5 units of packed red blood cells and one unit of platelets across hospitalizations
Shortly after hospitalizations	Developed new‐onset debilitating left hip pain. MRI showed findings concerning for a sclerotic bone lesion or atypical infection	CT‐guided biopsy pursued
CT‐guided femur biopsy	Confirmed osteonecrosis, likely related to prolonged systemic steroid use, with biopsy culture growing *Pseudomonas aeruginosa*	Admitted for IV antibiotics
Orthopedic evaluation	Orthopedic surgery found no indication for surgical intervention	Continued antibiotic management and nonoperative surveillance
Within a few months of osteomyelitis treatment	Hip pain has resolved	Continued multimodal pain management
Within 14 months of osteomyelitis diagnosis	The patient died	Comfort measures in hospice

Four years prior to diagnosis, the patient developed progressive bilateral lower‐extremity paresthesias with buttock, groin, and thigh pain. Despite serial evaluations, imaging demonstrated only mild degenerative spinal disease, and he was managed conservatively for multifactorial chronic pain with partial but transient relief.

Approximately 8 months before the onset of debilitating left hip pain, he presented with fatigue and generalized body aches and was found to have pancytopenia. Initial bone marrow biopsy was non‐diagnostic, and he was empirically treated with systemic corticosteroids for presumed autoimmune cytopenia. Persistent symptoms and worsening cytopenias later raised concern for evolving MDS, prompting rituximab therapy and prolonged corticosteroid exposure. His course was further complicated by recurrent hospitalizations for symptomatic anemia, gastrointestinal bleeding, and deep vein thrombosis (DVT) requiring inferior vena cava (IVC) filter placement.

Shortly thereafter, the patient developed severe left hip pain that significantly impaired ambulation and activities of daily living. Further evaluation demonstrated osteonecrosis complicated by superimposed *Pseudomonas aeruginosa* osteomyelitis. He was treated medically with prolonged antimicrobial therapy and nonoperative surveillance per orthopedic surgery recommendation.

### 2.2 Investigation and Treatment

#### 2.2.1 Imaging Course

Early lumbar MRI demonstrated mild levoscoliosis and multilevel degenerative disc disease without high‐grade stenosis (Figure 1A,B). Interval imaging showed progressive L4–L5 degenerative change, with enlargement of a broad‐based disc protrusion and contact of the traversing L5 nerve roots (Figure 1C,D). Later imaging demonstrated further progression to moderate‐to‐severe bilateral lateral recess stenosis and moderate central canal stenosis at L4–L5, with only mild degenerative changes at the remaining lumbar levels (Figure 1E,F). However, imaging findings remained insufficient to fully explain the patient's worsening symptoms. MRI obtained during the current presentation revealed a confluent intramedullary marrow abnormality within the left proximal femur with marked peri‐trochanteric edema and bursal fluid distention, prompting tissue biopsy (Figure 2).

**FIGURE 1:**
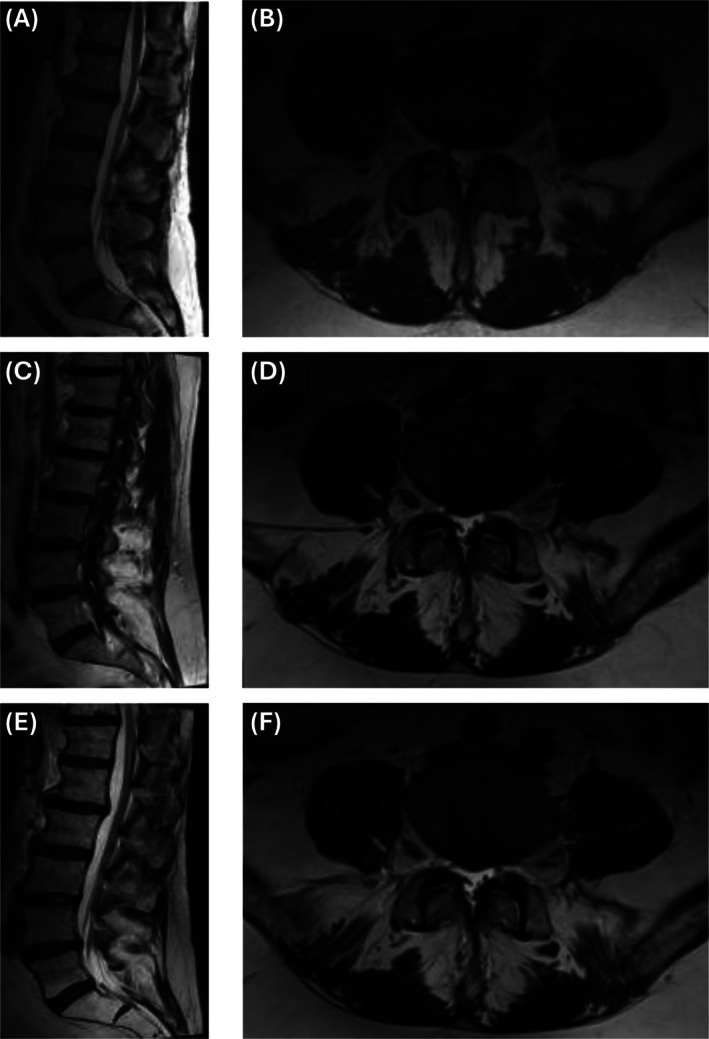
Serial lumbar spine MRI demonstrating interval progression of L4–L5 degenerative stenosis. Sagittal and axial T2‐weighted images obtained over approximately 3 years demonstrate progressive L4–L5 degenerative disease. Initial imaging (A, B) shows a broad‐based L4–L5 disc protrusion with mild bilateral lateral recess narrowing and no high‐grade central canal stenosis. Follow‐up imaging at approximately 2 years (C, D) demonstrates interval enlargement of the protrusion with contact of the bilateral traversing L5 nerve roots. Imaging at approximately 3 years (E, F) shows further progression to moderate‐to‐severe bilateral lateral recess stenosis and moderate central canal stenosis at L4–L5, with only mild degenerative changes at the remaining lumbar levels.

**FIGURE 2:**
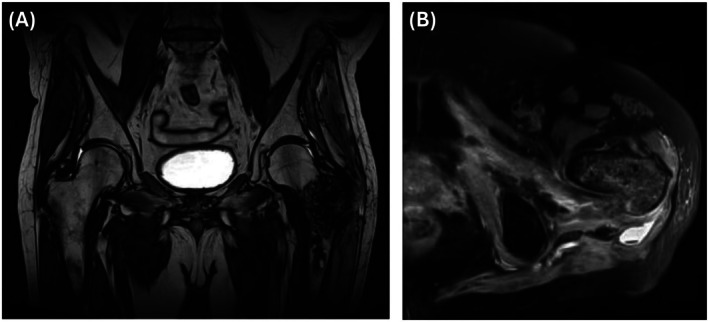
MRI of the pelvis showing a left proximal femoral marrow lesion. Coronal and oblique axial images (A, B) demonstrate an approximately 9‐cm intramedullary marrow signal abnormality in the left proximal femur, accompanied by extensive peritrochanteric edema and trochanteric bursal fluid.

#### 2.2.2 EMG/NCS

Electrodiagnostic testing demonstrated chronic neurogenic changes compatible with right S1 radiculopathy and possible chronic left L5/S1 involvement.

#### 2.2.3 Steroid Injections

Over several years, the patient underwent multiple image‐guided interventions, including intra‐articular hip corticosteroid injections, lumbar transforaminal epidural steroid injections, medial branch blocks, radiofrequency ablation, and piriformis injections. Two months prior to the development of the hip pain, patient had sacroiliac joint injections. These interventions provided temporary but incomplete pain relief.

#### 2.2.4 Bone Marrow Biopsy

Repeat bone marrow biopsy performed during evaluation of persistent pancytopenia demonstrated 5%–10% myeloblasts, multiple pathogenic mutations, and loss of the Y chromosome, concerning for evolving MDS. Prostate‐specific antigen remained undetectable, arguing against recurrent metastatic prostate cancer.

#### 2.2.5 High‐Dose Systemic Glucocorticoid Exposure

The patient received prolonged systemic corticosteroid therapy for presumed autoimmune cytopenia (eventual diagnosis of MDS), totaling well over 12‐g prednisone equivalent exposure during the clinical course, substantially increasing risk for steroid‐associated osteonecrosis and opportunistic infection.

Laboratory studies were notable for steroid‐associated hyperglycemia and leukopenia, although the latter was confounded by concurrent pancytopenia in the setting of evolving MDS. The patient also developed thrombotic complications, including DVT requiring IVC filter placement. No overt Cushingoid features, steroid‐induced myopathy, clinically significant electrolyte abnormalities, adrenal insufficiency, or other steroid‐related metabolic complications were documented during the clinical course.

#### 2.2.6 CT‐Guided Femur Biopsy

CT‐guided biopsy of the proximal femur demonstrated osteonecrosis and light growth of pan‐susceptible *Pseudomonas aeruginosa* . Follow‐up cross‐sectional imaging soon afterward demonstrated a lytic lesion in the same region (Figure 2). A repeat biopsy again identified *Pseudomonas aeruginosa* , localizing the infection to the femoral shaft rather than the femoral head.

#### 2.2.7 Antibiotic Therapy

The patient completed a six‐week course of IV piperacillin‐tazobactam and, because of persistent neutropenia associated with high‐risk MDS, transitioned to suppressive oral levofloxacin.

### 2.3 Surgical Decision‐Making and Follow‐Up

Orthopedic surgery deferred operative debridement in favor of close radiographic and clinical surveillance. At the time of evaluation, there was no evidence of mechanical instability, progressive collapse requiring reconstruction, drainable abscess, or failure of medical therapy that would clearly necessitate surgical intervention. In the setting of persistent neutropenia, evolving MDS, and superimposed infection, the risks of surgery, including poor wound healing, perioperative infectious complications, bleeding, and limited recovery after extensive debridement, were felt to outweigh the potential benefits of operative management. Surgical intervention would be reconsidered if he developed progressive infection despite antimicrobial therapy, worsening necrotic bone burden, abscess formation, structural compromise, or uncontrolled pain. Patient was then soon discharged.

At most recent follow‐up, the patient reported stable 3/10 pain while ambulating on suppressive antimicrobial therapy. The patient's subsequent clinical course was complicated by death 14 months after the diagnosis of osteomyelitis, attributed to progressive MDS.

## 3 Discussion

Patients living with long‐standing musculoskeletal or neuropathic pain are vulnerable to diagnostic overshadowing, a form of anchoring bias in which new symptoms are attributed to pre‐existing degenerative, radicular, or myofascial disease [16]. Our patient illustrates this hazard. His worsening left hip pain was initially interpreted in the context of known L4‐5 radiculopathy, sacroiliac dysfunction, and piriformis syndrome, delaying definitive hip‐focused evaluation until advanced osteonecrosis with superimposed infection was identified.

Systemic glucocorticoids remain the leading non‐traumatic trigger for femoral head osteonecrosis. A recent meta‐analysis of more than 23,000 steroid‐treated patients found an osteonecrosis incidence of approximately 6%–7%, with risk increasing substantially when cumulative prednisone‐equivalent doses exceed 10 g or daily doses exceed 40 mg [5, 17]. Our patient exceeded this cumulative exposure threshold while being treated for presumed autoimmune cytopenia that later evolved into MDS. Age‐related microvascular vulnerability, marrow dysplasia, persistent cytopenias, and B‐cell depletion from rituximab may have further impaired bone remodeling and host defense [18].

Clinical recognition of osteonecrosis is often delayed because early symptoms are subtle, frequently presenting as vague activity‐related pain, and plain radiographs can remain normal until substantial subchondral injury develops. MRI therefore underpins modern diagnosis, with meta‐analytic data demonstrating high sensitivity and specificity for detecting pre‐collapse femoral head lesions [19, 20]. Early identification matters because joint‐preserving measures, including activity modification, bisphosphonates, and core decompression, are most effective before structural failure, whereas late‐stage disease often progresses to arthroplasty despite aggressive management [21].

Superimposed infection in the setting of osteonecrosis is rare but has been described, particularly in immunocompromised hosts. Tan and Tan reported four immunosuppressed oncologic patients with early femoral head osteonecrosis and concomitant acute bacterial arthritis caused by enteric gram‐negative bacilli, emphasizing that symptoms and imaging may be nonspecific [22]. More recent reports have described osteonecrosis with concomitant septic arthritis after COVID‐19 infection and corticosteroid exposure, including bilateral hip involvement caused by *Pseudomonas aeruginosa* [23, 24]. Separately, Berthelot et al. reported *P. aeruginosa* osteomyelitis of both ischia, supporting the broader observation that pseudomonal pelvic bone infection is rare but clinically important [25]. Compared with these cases, the present case is distinct because infection localized to the proximal femur/femoral shaft rather than the femoral head or hip joint occurred in an elderly neutropenic patient with evolving MDS and had no clear source of seeding. Although the patient had undergone prior image‐guided pain procedures, including sacroiliac‐region steroid injections, the temporal and anatomic relationship to the femoral shaft infection was not definitive.

This case also highlights the importance of tissue diagnosis. MRI findings in osteonecrosis, osteomyelitis, and neoplastic marrow involvement can overlap, particularly in immunocompromised patients. CT‐guided biopsy was therefore decisive, confirming osteonecrosis while also identifying pan‐susceptible *P. aeruginosa* . This redirected management from elective orthopedic intervention alone toward culture‐directed antimicrobial therapy. Although surgical debridement is often considered in chronic osteomyelitis, operative management must be individualized. In our patient, the absence of mechanical instability, progressive collapse requiring reconstruction, drainable abscess, or failure of medical therapy favored nonoperative management. Given persistent neutropenia and evolving MDS, the risks of debridement, including poor wound healing, perioperative infection, bleeding, and impaired recovery, were felt to outweigh the immediate benefits. He was therefore treated with 6 weeks of IV piperacillin‐tazobactam followed by suppressive oral levofloxacin during ongoing cytopenia.

Several clinical lessons emerge from this case. First, any qualitative change in chronic pain warrants re‐evaluation rather than reflexive escalation of analgesics. Second, clinicians should maintain a low threshold for hip MRI in patients with prolonged systemic steroid exposure, hematologic malignancy, or immunosuppression. Third, suspicious marrow lesions in immunocompromised patients should be biopsied and cultured, as opportunistic infection can mimic tumor or sterile osteonecrosis. Finally, multidisciplinary coordination among hematology, orthopedics, infectious disease, pain management, and primary care is essential to balance antimicrobial therapy, steroid tapering, surgical risk, and joint‐preserving strategies.

This report has several limitations. As a single case, it cannot establish causality between corticosteroid exposure, osteonecrosis, and subsequent infection. The patient's evolving MDS, neutropenia, diabetes, recurrent hospitalizations, and prior pain procedures were important confounders that may have contributed to infection risk. The portal of entry for *P. aeruginosa* was not definitively identified. Finally, longer surveillance is needed to determine whether medical management alone will provide durable infection control or whether delayed surgical intervention will ultimately be required.

## Author Contributions

**Ji‐Hoon Lee:** conceptualization, data curation, formal analysis, investigation, methodology, project administration, validation, writing – original draft, writing – review and editing. **Elias Lai:** conceptualization, investigation, project administration, writing – original draft, writing – review and editing. **Jerry Markar:** conceptualization, investigation, methodology, resources, supervision, validation, writing – original draft, writing – review and editing. **Bi Mo:** conceptualization, investigation, methodology, resources, supervision, writing – original draft, writing – review and editing.

## Funding

The authors have nothing to report.

## Ethics Statement

Per UCLA policy, a case report that complies with HIPPA does not require IRB approval.

## Consent

Written informed consent was obtained from the patient for the publication of this case report and accompanying images. A copy of the signed consent is available for review upon request.

## Conflicts of Interest

The authors declare no conflicts of interest.

## Data Availability

Data and materials utilized to prepare for this case report were drawn from the patient's electronic medical records. Patient's protected health information is not available for disclosure per HIPPA.

## References

[1] A. Z. Ansari , D. U. Patel , S. Desai , A. Manawa , S. Patibandla , and K. Kratz , “Orthopedic Challenges in Avascular Necrosis Management: A Case Report of Surgical Complications and Multidisciplinary Care,” Cureus 16 (2024): e57629, 10.7759/cureus.57629. 38707141 PMC11069596

[2] A. Buddhiraju , H. S. Khanuja , V. Hegde , S. B. Sequeira , M. A. Mont , and L. C. Jones , “Epidemiology, Management, and Systematic Review of Surgical Trends for Patients Who Have Osteonecrosis of the Femoral Head,” Journal of Arthroplasty 40 (2025): S112–S119.e1, 10.1016/j.arth.2025.06.002.40490143

[3] M. J. Lespasio , N. Sodhi , and M. A. Mont , “Osteonecrosis of the Hip: A Primer,” Permanente Journal 23 (2019): 18–100, 10.7812/TPP/18-100. PMC6380478 30939270

[4] K. N. Shah , J. Racine , L. C. Jones , and R. K. Aaron , “Pathophysiology and Risk Factors for Osteonecrosis,” Current Reviews in Musculoskeletal Medicine 8 (2015): 201–209, 10.1007/s12178-015-9277-8. 26142896 PMC4596210

[5] V. Birla , A. Vaish , and R. Vaishya , “Risk Factors and Pathogenesis of Steroid‐Induced Osteonecrosis of Femoral Head ‐ A Scoping Review,” Journal of Clinical Orthopaedics and Trauma 23 (2021): 101643, 10.1016/j.jcot.2021.101643. 34722150 PMC8531658

[6] W. Konarski , T. Poboży , K. Konarska , et al., “Osteonecrosis Related to Steroid and Alcohol Use—An Update on Pathogenesis,” Health 11 (2023): 1846, 10.3390/healthcare11131846. PMC10340773 37444680

[7] Q. Cui , W.‐L. Jo , K.‐H. Koo , et al., “ARCO Consensus on the Pathogenesis of Non‐Traumatic Osteonecrosis of the Femoral Head,” Journal of Korean Medical Science 36 (2021): e65, 10.3346/jkms.2021.36.e65. 33724736 PMC7961868

[8] J. Hatzenbuehler and T. J. Pulling , “Diagnosis and Management of Osteomyelitis,” American Family Physician 84 (2011): 1027–1033. 22046943

[9] P. H. Johanson , “Pseudomonas Infections of the Foot Following Puncture Wounds,” JAMA 204 (1968): 262–264, 10.1001/jama.1968.03140160072024. 4384798

[10] P. H. Chandrasekar and A. P. Narula , “Bone and Joint Infections in Intravenous Drug Abusers,” Reviews of Infectious Diseases 8 (1986): 904–911, 10.1093/clinids/8.6.904. 3099362

[11] B. A. Lipsky , “Osteomyelitis of the Foot in Diabetic Patients,” Clinical Infectious Diseases 25 (1997): 1318–1326, 10.1086/516148. 9431370

[12] C. C. Blyth , L. Gomes , T. C. Sorrell , M. da Cruz , A. Sud , and S. C.‐A. Chen , “Skull‐Base Osteomyelitis: Fungal vs. Bacterial Infection,” Clinical Microbiology and Infection 17 (2011): 306–311, 10.1111/j.1469-0691.2010.03231.x. 20384699

[13] N. B. Shah , D. R. Osmon , J. M. Steckelberg , et al., “Pseudomonas Prosthetic Joint Infections: A Review of 102 Episodes,” Journal of Bone and Joint Infection 1 (2016): 25–30, 10.7150/jbji.15722. 28529849 PMC5423557

[14] S. P. Mironov , A. V. Tsiskarashvili , and D. S. Gorbatiuk , “Chronic Post‐Traumatic Osteomyelitis as a Problem of Contemporary Traumatology and Orthopedics (Literature Review),” Go 25 (2019): 610–621, 10.18019/1028-4427-2019-25-4-610-621.

[15] M. Li , Z. Shao , H. Zhu , and Y. Zhang , “The Diagnosis and Treatment of Septic Hip With Osteonecrosis of the Femoral Head,” Journal of Orthopaedic Surgery and Research 19 (2024): 46, 10.1186/s13018-023-04518-6. 38195515 PMC10775663

[16] R. Murani and R. H. K. Manocha , “Avoiding Anchoring Bias in Unexplained Chronic Pain: An Unexpected Diagnosis of Synovial Osteochondromatosis,” BML Case Reports 14 (2021): e240462, 10.1136/bcr-2020-240462. PMC8030675 33827875

[17] M. A. Mont , R. Pivec , S. Banerjee , K. Issa , R. K. Elmallah , and L. C. Jones , “High‐Dose Corticosteroid Use and Risk of Hip Osteonecrosis: Meta‐Analysis and Systematic Literature Review,” Journal of Arthroplasty 30 (2015): 1506–1512.e5, 10.1016/j.arth.2015.03.036. 25900167 PMC7127809

[18] M.‐J. Javelot , J. Sergheraert , S. Agbo‐Godeau , et al., “Rituximab as a Trigger Factor of Medication‐Related Osteonecrosis of the Jaw. A Case Report,” Journal of Stomatology, Oral and Maxillofacial Surgery 121 (2020): 300–304, 10.1016/j.jormas.2019.06.009. 31301390

[19] Expert Panel on Musculoskeletal Imaging , A. S. Ha , E. Y. Chang , et al., “ACR Appropriateness Criteria Osteonecrosis: 2022 Update,” Journal of the American College of Radiology 19 (2022): S409–S416, 10.1016/j.jacr.2022.09.009. 36436966

[20] W. Konarski , T. Poboży , A. Śliwczyński , et al., “Avascular Necrosis of Femoral Head—Overview and Current State of the Art,” International Journal of Environmental Research and Public Health 19 (2022): 7348, 10.3390/ijerph19127348. 35742595 PMC9223442

[21] A. Lohiya , N. Dhaniwala , U. Dudhekar , S. Goyal , and S. K. Patel , “A Comprehensive Review of Treatment Strategies for Early Avascular Necrosis,” Cureus 15 (2023): e50510, 10.7759/cureus.50510. 38226130 PMC10788237

[22] T. Tan and S. Tan , “Concomitant Early Avascular Necrosis of the Femoral Head and Acute Bacterial Arthritis by Enteric Gram‐Negative Bacilli in Four Oncologic Patients,” Smedj 54 (2013): e108–e112, 10.11622/smedj.2013065. 23716162

[23] A. Parameswaran , M. A. Mohammed , D. Gautam , R. Malhotra , S. Apsingi , and K. K. Eachempati , “Osteonecrosis With Concomitant Septic Arthritis of Bilateral Hips Following Severe COVID‐19 Infection: A Case Report,” Journal of Orthopaedic Case Reports 15 (2025): 65–69, 10.13107/jocr.2025.v15.i02.5230. PMC11823863 39957919

[24] M. V. Ardakani , S. Parviz , E. Ghadimi , et al., “Concomitant Septic Arthritis of the Hip Joint and Femoral Head Avascular Necrosis in Patients With Recent COVID‐19 Infection: A Cautionary Report,” Journal of Orthopaedic Surgery and Research 17 (2022): 302, 10.1186/s13018-022-03192-4. 35668523 PMC9168355

[25] J.‐M. Berthelot , S. Varin , F. Caillon , A. Prost , and Y. Maugars , “ *Pseudomonas aeruginosa* Osteomyelitis of Both Ischia : A Case Report,” JBJS 84 (2002): 441–444. 10.2106/00004623-200203000-00017 11886916

